# A Randomized Controlled Trial to Determine the Effects of Curcumin and Epigallocatechin-3-Gallate Supplementation on Serum Brain-Derived Neurotrophic Factor and Mood Disturbance in Adults

**DOI:** 10.3390/nu18050855

**Published:** 2026-03-06

**Authors:** Aidan M. Cavanah, Laura A. Robinson, Madison M. Aguilar, Elaine F. Molaison, Michael W. Greene, Michael D. Roberts, Andrew D. Fruge

**Affiliations:** 1 College of Nursing, Auburn University, Auburn, AL 36849, USA; ldelvers@purdue.edu (L.A.R.); adf0003@auburn.edu (A.D.F.); 2 Department of Nutritional Sciences, Auburn University, Auburn, AL 36849, USA; elaine.molaison@auburn.edu (E.F.M.); mwg0006@auburn.edu (M.W.G.); 3 School of Kinesiology, Auburn University, Auburn, AL 36849, USA; mlm0139@auburn.edu (M.M.A.); mdr0024@auburn.edu (M.D.R.)

**Keywords:** epigallocatechin-3-gallate (EGCG), curcumin, mood disorders, brain-derived neurotrophic factor (BDNF), depression, anxiety, stress

## Abstract

**Background/Objectives**: Mood disorders like depression, anxiety, and stress have increased steadily among adults, with growing interest in non-pharmaceutical treatments to improve symptomology. Epigallocatechin-3-gallate (EGCG) and curcumin are polyphenols with evidence to support their positive impacts on mood disorder symptomology and potential mood-associated biomarkers like brain-derived neurotrophic factor (BDNF). This study examined the effects of combined EGCG and curcumin supplementation on mood disturbance symptomology and serum brain-derived neurotrophic factor in adults. **Methods**: An 8-week randomized double-blinded placebo-controlled trial was conducted in adults (*n* = 64, 18–50 years old). Participants were randomized to a supplement group (*n* = 32; 350 mg EGCG and 1330 mg curcumin daily) or a matched placebo group (*n* = 32). Mood disturbance (DASS-21, GAD-7), sleep disturbance (GSAQ), and physical activity (IPAQ) were assessed at baseline, Week 4, and Week 8. Anthropometric measures, 24 h diet recalls, and fasted blood samples for serum BDNF were collected at baseline and Week 8. A multivariate ANOVA evaluated primary outcomes (DASS-21 composite score and BDNF), followed by repeated measures ANOVA for secondary outcomes (*p* < 0.05). **Results**: Significant improvements were observed across all participants for mood (DASS-21 composite and subscales, GAD-7, *p* < 0.001 for all), sleep (*p* < 0.001), and physical activity (*p* < 0.01), with no significant difference between supplement and placebo groups. Mean serum BDNF increased in both groups, but neither were statistically significant with no group-by-time interactions. Sugar intake (g/kg body weight) was positively correlated with mood symptoms at Week 8 in the supplement group. Baseline fruit and vegetable intake was associated with mood symptom severity at select time points; however, dietary changes during the intervention were not significantly related to changes in mood outcomes. **Conclusions**: Combined EGCG and curcumin supplementation did not show additional benefits beyond placebo for mood disturbance or serum BDNF over eight weeks. Observed improvements across both groups suggest that behavioral or lifestyle factors may play a larger role in short-term mood improvements than supplementation alone.

## 1. Introduction

Mood disorders are notable disruptions in emotions that occur internally but can impact a person’s behavior externally [1]. Similarly, mental health disorders impact a person’s thinking, feelings, mood, and behavior [2]. Mental health conditions in young adults and college students have been on the rise, with the most recent report from the National Healthy Minds Study finding > 60% of college students met criteria for one or more mental health conditions, a 50% increase since 2013 [3]. Global epidemiological studies indicate the prevalence of mood disorders was more than 40% in the “emerging adulthood” population, which spans 18–29 years of age [4]. Importantly, the rise in mental health disorders is a global concern. In 2018, the World Health Organization implemented the first stage of the WHO World Mental Health International College Student project. This was a series of surveys for first-year college students from 19 colleges across eight countries, including the United States. Of the nearly 14,000 respondents, 31% screened positive for one or more of the Diagnostic and Statistical Manual of Mental Disorders, fourth edition (DSM-IV) mental disorders: major depression, mania/hypomania, generalized anxiety disorder, panic disorder, and alcohol or substance use disorder [5]. Major depression has a lifetime prevalence of 5–17%, with adults showing an annual prevalence rate of 7.1% in the United States [6].

Pathophysiology has revealed that one of the primary risk factors for depression is chronic stress due to glucocorticoid effects on the hypothalamic–pituitary–adrenal (HPA) axis [7]. Neuronal plasticity also plays an important role in mood disorder pathophysiology, with plasticity impairment showing predisposition to its progression and development [8]. One protein that has been shown to impact both neuronal plasticity and the HPA axis is brain-derived neurotrophic factor (BDNF). BDNF is a neurotrophin that modulates brain neuroplasticity and has been widely studied in its effect on psychiatric disorders [9]. BDNF exerts neuroprotective and neuroactivating properties on both excitatory and inhibitory neurons, with high concentrations found in the hippocampus and frontal cortex [10]. Lower serum BDNF levels have been observed in patients with major depressive disorder (MDD) when compared to healthy controls [11]. These studies support the ‘neurotrophin hypothesis of depression’, which states that reduced levels of BDNF in the brain contribute to cell atrophy in the hippocampus and prefrontal cortex [12]. Increases in BDNF have been associated with improved depressive symptoms in several studies, supporting the hypothesis that altering BDNF may precede improved mental health [13].

Studies show that people with mental health disorders, especially depression, are seeking and using complementary and alternative medicine (CAM) treatment methods at increased rates in comparison to other health conditions [14]. While psychopharmacological and non-drug therapies can be effective, 30–40% of patients with major depression eventually become resistant to treatment due to various complications, ranging from medical comorbidities to poor adherence to therapy [15]. Due to its limited prevalence, many Western medical practitioners have a knowledge gap regarding CAM treatments, which may limit discussion of alternative treatment options [16]. This highlights the need for evidence-based resources and guidelines from reputable research trials surrounding CAM therapy for mood disorders like depression. The National Institute of Health defines CAM as therapies not usually included in conventional Western medical practice, with interventions focused on the use of other treatments like herbal substances and non-prescribed supplements [17]. Considering that nearly 60% of people with depression avoid seeking any medical support, alternative treatments for mood disorders like depression, anxiety, and stress need to be thoroughly researched [18].

Polyphenols are bioactive compounds occurring as secondary metabolites in plants [19]. Polyphenolic compounds like Ginkgo biloba and those found in green tea are known to be involved in brain plasticity, mood, depression, and cognition, showing success in lowering the incidence of psychiatric disorders [20]. Green tea comes from the *Camellia sinensis* plant and is high in a subgroup of polyphenolic compounds belonging to the flavonoid family known as catechins. The structural characteristics of these catechins are highlighted by the presence of a benzopyran structure containing at least one aromatic ring [21]. There are four main catechins that comprise the majority of the bioactive weight, differing in side groups that alter their physiological interactions. The largest and most studied of the four is (-)epigallocatechin-3-gallate (EGCG), which contains a benzenediol ring, a tetrahydropyran moiety, a pyrogallol ring, and a galloyl group [22]. Rodent studies have shown that EGCG aids in alleviating depressive symptoms without toxic side effects through the promotion of regulatory pathways like autophagy [23]. In other chronic unpredictable mild stress (CUMS) mouse studies, EGCG effectively decreased stress biomarkers like interleukin-1β while increasing mRNA expression of BDNF in the hippocampal regions [24].

A human double-blinded placebo-controlled crossover study found that supplementation with 300 mg EGCG was associated with increased self-rated calmness and reduced stress [25]. Much of the current literature surrounding the use of green tea and EGCG uses healthy adults as the study population. One clinical trial found that powdered green tea with a caffeine/EGCG (CE) to theanine/arginine (TA) mole ratio from 3.9 to 4.7 significantly decreased State-Trait Anxiety Inventory scores and depressive tendencies post-intervention [26]. Continued caffeine ratio changes have also shown that low-caffeine green tea significantly lowers stress and reduces post-stress salivary amylase activity [27]. Forms of green tea have also been studied in human trials, with 3 g of matcha tea significantly reducing anxiety compared to a placebo over a 15-day period [28]. A 5-week study using green tea powder at 400 mg, 3x/day, found significant improvements in depressive symptoms compared to placebo [29]. A study that used patients diagnosed with schizophrenia disorder, schizoaffective disorder, or bipolar disorder observed that supplementation with 150 mg EGCG capsules for 8 weeks found no significant psychiatric effects compared to placebo. Both groups saw significant decreases in selective mood scales [30]. The current array of human clinical trials uses various forms of green tea, not just pure EGCG, but lacks extensive clinical trial duration.

Curcumin is the main curcuminoid and bioactive compound in turmeric, which comes from the *Curcuma longa* plant. It has been utilized for centuries to treat a variety of diseases and conditions [31]. The monoamine deficiency theory is thought to explain a potential cause for major depression, as it states that the decrease in monoamines like serotonin, noradrenaline, and dopamine in the central nervous system is core to the pathophysiology of depression [32]. Studies in mice have shown reversed depressive-like behavior due to chronic stress, increased serotonergic and dopaminergic transmission, as well as the inhibition of monoamine-oxidase, which breaks down monoamines, following curcumin supplementation [33]. Various studies have found similar results with dose-dependent curcumin increasing serotonin and dopamine in the frontal cortex and hippocampus of rats while again inhibiting monoamine oxidase enzymes [34,35]. Additionally, curcumin has been found to interact with mood regulatory signaling pathways like cyclic adenosine monophosphate (cAMP), response element-binding protein (CREB) pathway, and BDNF pathways [36]. Serotonin, or 5-hydroxytryptamine (5-HT), signaling disruption in the brain is also believed to be involved in depression pathophysiology. Studies show BDNF acts as a modulator of this 5-HT system, leading to the understanding that dysregulation in this 5-HT-BDNF interaction can cause neuropsychiatric and behavioral abnormalities [37]. Polyphenolic compounds like EGCG have demonstrated the alleviation of depressive symptoms by increasing 5-HT levels in rats’ hippocampal region [38]. Neuroprotective effects of polyphenols stem from their anti-inflammatory and antioxidant properties, including the modulation of neurotransmitter systems and neurodegeneration prevention [39]. In a preclinical study, polyphenols found in curcumin suppressed the decrease in BDNF levels induced by exogenous corticosterone administration [40]. Similarly, EGCG exhibited neuroprotective effects via BDNF in mouse models [41]. A recent systematic review of 48 studies on dietary interventions and BDNF levels found four of the 11 polyphenol-specific studies observed significant increases in BDNF concentrations [42].

Many of the studies that explore EGCG and curcumin’s impact on mood disorders like depression are rodent studies, which highlights a need for solid, foundational human-based research. Therefore, the purpose of this study was to explore the impact of supplementation with EGCG and curcumin on mood disturbance and serum BDNF in adults. The primary aim is to measure change in DASS-21 composite scores and serum BDNF neurotrophic factor concentrations. Secondary aims include assessing changes in GAD-7, GSAQ, IPAQ, AST, ALT, 24 h diet recalls, and adherence to the supplementation. The intervention group was instructed to take 1 tablet (350 mg) of EGCG and 2 tablets (1330 mg total) of curcumin daily for 8 weeks while avoiding the concurrent consumption of green tea and turmeric. The placebo group was instructed to take 1 tablet of the EGCG placebo and 2 tablets of the curcumin placebo daily for 8 weeks while also avoiding the consumption of green tea and turmeric. Notably, the participants as well as the study staff were blinded to treatment regimens, and this is discussed in greater detail in the following paragraphs. We hypothesize that consumption of 350 mg/day of epigallocatechin-3-gallate (EGCG) and 1330 mg/day of curcumin for 8 weeks will significantly improve mood disorder symptomology scores and increase serum brain-derived neurotrophic factor.

## 2. Materials and Methods

### 2.1. Ethical Approval and Participant Eligibility

This double-blinded randomized placebo-controlled trial was conducted with prior review and approval from the Auburn University Institutional Review Board and in accordance with the most recent revisions of the Declaration of Helsinki and registered as a clinical trial (NCT06531863) on 16 July 2024. Participants were screened for depressive symptoms using the depression, anxiety, stress scale (DASS-21) beginning in October 2024. Inclusion criteria were ages 18–50, a DASS-21 Depression subscale score of >9/21 indicating moderate to severe symptomology, no change in medications or supplements over the past 3 months, and can read and speak English. Exclusion criteria were currently consuming green tea or curcumin daily, currently pregnant, nursing, or trying to become pregnant, or currently diagnosed with a perimenopausal disorder. Following verbal and written consent, eligible participants were enrolled in the study and completed procedures outlined below. Formal psychiatric diagnoses were not established, and information regarding duration of symptoms or prior treatment history was not collected. The sample was predominantly female.

### 2.2. Procedure

Participants reported to the School of Kinesiology at Auburn University, where, once screened, informed consent was received and medical history was obtained. Participants were then randomly allocated to either the intervention or the control group. At baseline, anthropometric data (height, weight, bone mass, lean mass, and body fat percentage) were collected via bioelectrical impedance. A 24 h dietary recall of the day prior to the blood draw and one weekend or weekday (depending on the day of the initial visit) was performed by a registered dietitian. An 8 h fasted 5 mL venipuncture blood draw was performed, and the following questionnaires were completed via Qualtrics, DASS-21, GAD-7, IPAQ, and GSAQ. Midway through the intervention at Week 4, a link to the same questionnaires was sent via Qualtrics for the participants to complete again (DASS-21, GAD-7, IPAQ, and GSAQ). At Week 8, participants completed the post-intervention assessment, which included everything previously completed at their baseline visit, anthropometrics, 24 h dietary recalls, an 8 h fasted blood draw, and completion of the same four questionnaires (DASS-21, GAD-7, IPAQ, and GSAQ) via Qualtrics. An eight-week intervention period was chosen based on previous randomized controlled trials employing similar supplementation durations and reporting mood-related outcomes using comparable methodologies [30,43,44].

### 2.3. Sample Size and Power

To determine the minimum sample size required for detecting multivariate effect across the primary outcomes of DASS-21 composite scores and serum BDNF levels, an a priori power analysis was conducted. The power analysis used the G*Power version 3.1.9.7 FAUL for sample size estimation, based on data from a 2018 meta-analysis (*n* = 57), which compared various interventions, including DASS-21 on depression [45]. The effect size in this study was 0.378, considered to be medium using Cohen’s criteria [46]. With a significance criterion of α = 0.05 and power = 0.80, the minimum sample size needed with this effect size is *n* = 57 for a multivariate effect using MANOVA. Our goal was to enroll 64 participants in the trial, as with this number of participants, the trial will still be fully powered with 10% attrition.

### 2.4. Randomization

Participants were randomized using the Sealed Envelope randomization application [47]. Randomization into the intervention and placebo groups was done via stratification by gender (male or female) and DASS-21 depression subscale scores (moderate [10,11,12,13,14,15,16,17,18,19,20] or severe [21+]) in blocks of four. Dummy codes corresponding to a combination of gender and depression subscale scores (i.e., male-severe, female-moderate, etc.) were placed on bottles coded as intervention or placebo by a research staff member and placed in a brown paper bag. A different research staff member provided the dummy-coded bag to participants and was blind to their randomized group. Directions for consumption were printed on the bottle labels and discussed with the participants.

### 2.5. Supplementation

Participants in the intervention group received 350 mg EGCG pills, of which 48 mg were calcium as dibasic calcium phosphate. They also received 665 mg curcumin pills, of which curcuminoids comprised 95% (630 ng). They were instructed to consume one pill of EGCG and two pills of curcumin daily with their first meal for the eight-week intervention. The placebo pills consisted of maltodextrin. To ensure optimal adherence, participants confirmed supplement intake by responding to an automated text message from the Emitrr messaging software (Emitrr, Lewes, DE, USA; https://emitrr.com/) sent every morning at 7 a.m. for the duration of the intervention.

### 2.6. Anthropometric Measurements

Anthropometric measurements were taken at baseline and Week 8 (post-intervention). All participants were measured without shoes, socks, or electrical devices in their pockets. A free-standing stadiometer was used to obtain height, while body composition was assessed with the multi-frequency analyzer TANITA bioelectrical impedance analysis (BIA) scale (BC-568 Innerscan Segmental Body Composition Monitor, Arlington Heights, IL, USA). Weight, lean body mass, bone mass, and body fat percentage were recorded for all participants.

### 2.7. Dietary Habits

The dietary recalls of all participants were recorded and performed by the research team of registered dietitians. The registered dietitians conducted 24 h dietary recalls that included food and beverages with corresponding portion sizes and weights the day prior to the blood draw and a previous weekend or weekday, depending on the day of the baseline meeting. The lead research dietitian then logged all recalls into the Nutrition Data System for Research (NDSR; Nutrition Coordinating Center, University of Minnesota, Minneapolis, MN, USA) dietary analysis program, which quantifies and analyzes the nutrient composition of the participants’ dietary intake [48]. Healthy Eating Index (HEI) scores from NDSR for total fruit and total vegetable intake were taken from baseline recalls and included as covariates to account for potential confounding effects of baseline dietary polyphenol intake. This approach enabled control for baseline dietary variability while evaluating the effects of time and treatment group on psychological outcomes.

### 2.8. Depression

Depressive symptomology and severity were measured using the twenty-one-item depression, anxiety, stress scale (DASS-21) [49]. This seven-item questionnaire contains three domains with a 4-point Likert scale referencing the level of applicability to the reader, respectively. Severity is determined by the sum of the subscale domain scores. Good internal consistency and validity have been reported with this questionnaire, especially with the depression domain, which is supported by a Cronbach alpha range of α = 0.761 to α = 0.906 [50].

### 2.9. Anxiety

Anxiety symptoms were assessed using the Generalized Anxiety Disorder questionnaire (GAD-7), a seven-item tool that employs a Likert scale ranging from 0 to 3. The GAD-7 has demonstrated high internal consistency, with a Cronbach α = 0.92, and strong test–retest reliability, indicated by an intra-class correlation of 0.83 [51].

### 2.10. Sleep

Sleep disorder symptoms were evaluated using The Global Sleep Assessment Questionnaire (GSAQ), an eleven-item measure with response options of “never,” “sometimes,” “usually,” and “always.” Participants are prompted to select the response that best reflects their experiences over the past four weeks. Studies by Roth et al., indicate that the GSAQ demonstrates reliability, validity, sensitivity, and specificity comparable to more extensive sleep assessment tools [52].

### 2.11. Physical Activity

Physical activity levels were measured using the International Physical Activity Questionnaire Short Form (IPAQ-SF), a seven-item instrument relying on participants’ recall of the previous seven days. The IPAQ-SF has shown strong test–retest reliability, with a Spearman correlation of *p* = 0.8 and criterion validity of *p* = 0.30, demonstrating its comparability with other self-reported physical activity questionnaires [53].

### 2.12. Serum Brain-Derived Neurotrophic Factor

Following an eight-hour fast, 1–7 mL of whole blood was collected using BD Vacutainer™ Venous Blood Collection Tubes: Vacutainer Plus™ Plastic Serum Tubes, Silicone Coated, with Conventional Stopper [54]. Following collection, tubes were inverted 6–8 times and set upright in a tube holder at room temperature to allow clotting. Samples were then centrifuged at 2000 rpm at 22 °C for 25 min or until adequate serum separation occurred. After centrifugation, the serum was pipetted into two 1 mL microtubes labeled with the corresponding participant identification number, date of blood draw, and visit number (baseline or post-intervention) of the participant. The microtubes were then stored at −80 °C until they were analyzed for BDNF concentrations. Serum brain-derived neurotrophic factor (BDNF) concentrations were determined using a Catchpoint SimpleStep human BDNF ELISA kit (Product Code: ab229395, Abcam Inc., Waltham, MA, USA). All samples were analyzed in duplicate following the manufacturer’s protocol. A SpectraMax i3x microplate reader measured absorbance at 450 nm. The assay detection range spanned 35–2000 pg/mL, with a reported sensitivity of 8 pg/mL and intra- and inter-assay coefficients below a 10% variation.

### 2.13. Statistics

Data analyses were performed using SPSS Version 29.0 (IBM Corp, Armonk, NY, USA) and GraphPad Prism (Version 10.1; San Diego, CA, USA). Independent samples *t*-tests were used to assess baseline equivalence between the intervention and placebo groups on mood disorder questionnaires and serum BDNF. The primary outcome to examine the effect of supplementation on Week 8 outcomes was done using multivariate analysis of variance (MANOVA), followed by univariate ANOVAs exploring between-group differences in outcome measures. Two-way repeated measures ANOVA assessed within-subject changes over time for mood and serum BDNF. Consistent with prior randomized controlled trials employing longitudinal repeated measures ANOVA to assess treatment by time effects [30,43,55,56], the statistical approach utilized was considered appropriate for the present dataset.

Changes between time points were measured with Bonferroni-adjusted pairwise comparisons, with linear and quadratic trend analyses assessing patterns of change for GAD-7 and GSAQ. Categorical changes in physical activity were examined using nonparametric analyses. Overall shifts in IPAQ activity classifications were evaluated with Friedman tests, followed by post hoc Wilcoxon signed-rank tests applying Bonferroni adjustments. Chi-square analyses were used to compare IPAQ category distributions across time points. Changes in dietary intake variables were analyzed using repeated measures ANOVA, while independent samples t-tests compared intake between intervention and placebo groups. ANCOVA models adjusting for baseline Healthy Eating Index fruit and vegetable intake were used to control for background polyphenol intake. Associations between sugar intake normalized to body weight (g/kg) and mental health outcomes were evaluated using Pearson correlation coefficients. Additionally, a standard multiple linear regression model was applied to examine whether baseline DASS-21 composite, GAD-7, or GSAQ scores predicted changes in serum BDNF concentrations.

## 3. Results

### 3.1. Participant Characteristics and Compliance

**Figure 1:**
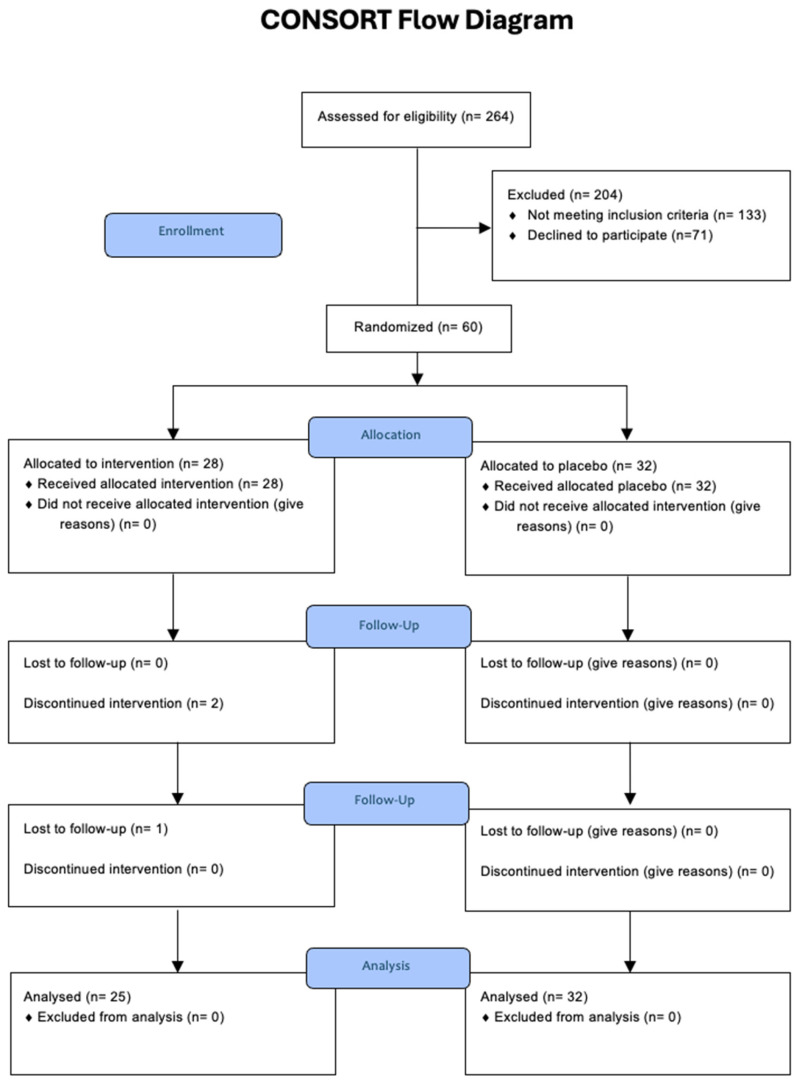
CONSORT flow diagram.

The CONSORT flow diagram (Figure 1) shows participant accrual and study retention. A total of 264 individuals were assessed for eligibility, with 204 of those being excluded (133 for not meeting inclusion criteria and 71 declining to participate). Sixty participants met the eligibility criteria, including DASS-21 Depression subscale scores of >9/21, and were contacted for participation in the study. Of the 60 participants who were eligible, 60 were included in the study, being randomized into intervention (*n* = 28) and placebo (*n* = 32) groups. There was a withdrawal of two participants in the intervention group before the Week 4 midpoint assessment, one due to adverse reactions to the supplement and the other due to religious conflicts. A third participant withdrew before the Week 8 post-intervention assessment from the intervention group due to loss of contact. In total, 57 participants completed the 8-week intervention and were included in final analyses (intervention group: *n* = 25; placebo group: *n* = 32).

Demographic and baseline variables for the included participants are outlined in Table 1. No significant baseline differences were found between the intervention and placebo groups regarding age (*p* = 0.235), gender (*p* = 0.886), BMI (*p* = 0.713), or ethnicity (*p* = 0.726).

Baseline comparability between randomized groups was evaluated using independent samples t-tests. No significant baseline differences were observed between the intervention and placebo groups for measures of DASS-21 depression, t(58) = −1.41, *p* = 0.163; DASS-21 stress, t(58) = 1.27, *p* = 0.209; DASS-21 anxiety, t(58) = 0.088, *p* = 0.930; GAD-7, t(53.11) = 0.278, *p* = 0.782; IPAQ, t(56) = 1.26, *p* = 0.214; GSAQ aggregate, t(58) = 0.897, *p* = 0.373; DASS-21 composite, t(58) = −0.133, *p* = 0.895; or serum BDNF, t(56) = −0495, *p* = 0.622. This suggests successful randomization, as both groups were comparable at the start of the intervention.

### 3.2. Primary Outcomes

To examine primary outcomes, a multivariate analysis of variance (MANOVA) was performed on serum BDNF and DASS-21 composite scores at Week 0 and Week 8. A significant main effect of time was observed, Wilks’ L = 0.523, *F*(2, 50) = 22.80, *p* < 0.001, partial h^2^ = 0.477, indicating the overall changes across time were significant.

**Figure 2:**
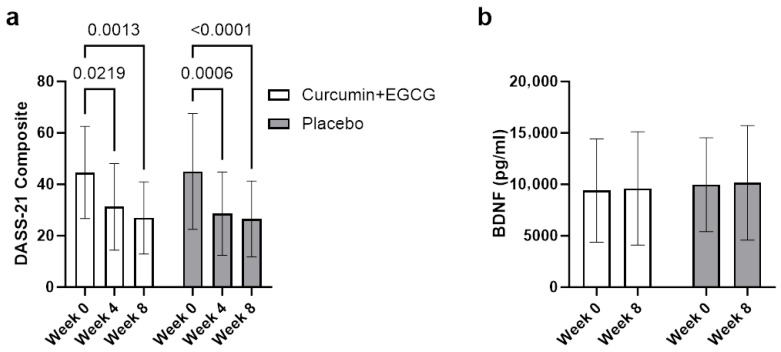
Changes in DASS-21 composite scores (**a**) and serum brain-derived neurotrophic factor—BDNF (**b**) from Week 0 to Week 8 by treatment group. Both groups showed significant reductions in mood symptoms over time (*p* < 0.001) and an increase in serum BDNF, but not to a statistically significant degree, with no significant group × time interactions for either outcome (*p* > 0.28), indicating similar changes across the intervention and placebo groups. Values represent means ± standard deviations (SD).

However, no significant multivariate interaction between time and treatment group was observed, Wilks’ L = 0.998, *F*(2, 50) = 0.04, *p* = 0.962, partial h^2^ = 0.002. Follow-up univariate analyses revealed a significant reduction in DASS-21 composite scores over time (Figure 2a), *F*(1, 51) = 46.23, *p* < 0.001, partial h^2^ = 0.475, whereas serum BDNF concentrations did not significantly change over time, *F*(1, 51) = 0.03, *p* = 0.857, partial h^2^ = 0.001 (Figure 2b).

#### 3.2.1. DASS-21 Composite Score

To assess the effect of supplementation on DASS-21 composite scores, a two-way repeated measures ANOVA was conducted. Mood disturbance significantly improved over the 8-week intervention with a significant main effect of time being observed, *F*(2, 110) = 35.62, *p* < 0.001, partial h^2^ = 0.393. No significant interaction between time and treatment group was found, *F*(2, 110) = 0.25, *p* = 0.780, partial h^2^ = 0.005, indicating no significant change in mood disturbance between the intervention and placebo groups over time (Figure 2a). There was also no significant between-group difference in overall DASS scores, *F*(1, 55) = 0.06, *p* = 0.811. To be of note was a clinically meaningful reduction in mood symptoms observed with the average reduction in DASS-21 composite scores in both groups being statistically significant (M = 18.2, *p* < 0.001). Significant reductions were seen after pairwise comparisons using Bonferroni corrections, from Week 0 to Week 4 (*p* < 0.001) and from Week 0 to Week 8 (*p* < 0.001), but not between Week 4 and Week 8 (*p* = 0.174).

#### 3.2.2. Serum Brain-Derived Neurotrophic Factor

Repeated measures ANOVA was conducted to evaluate the effect of time and treatment group on serum BDNF. No significant main effect of time was observed across participants from baseline to Week 8, indicating no significant change in BDNF concentrations, *F*(1, 51) = 0.03, *p* = 0.857, partial h^2^ =0.001 (Figure 2b). The interaction between time and group was also not significant in serum BDNF changes between intervention and placebo groups, *F*(1, 51) = 0.00, *p* = 0.987. No significant between-subjects effect of treatment group was observed for serum BDNF *F*(1, 51) = 0.33, *p* = 0.570, partial h^2^ = 0.006, indicating no overall difference in concentrations between groups across time points. Although mean serum BDNF concentrations did increase from baseline to Week 8 across all participants (M = 9707 pg/mL to M = 9895 pg/mL), the change was not statistically significant, *F*(1, 51) = 0.03, *p* = 0.857, and did not differ by treatment group.

Pearson correlation analyses examined whether changes in psychological and behavioral outcomes were related to changes in serum BDNF across the intervention. Change scores were determined as the difference between Week 8 and baseline values. No significant correlations were identified between changes in BDNF and changes in depression (r = 0.033, *p* = 0.817), anxiety (r = 0.079, *p* = 0.574), stress (r = 0.031, *p* = 0.825), DASS-21 composite (r = 0.050, *p* = 0.720), generalized anxiety (GAD-7; r = −0.037, *p* = 0.794), physical activity level (IPAQ; r = −0.092, *p* = 0.530), or sleep disturbance (GSAQ; r = −0.054, *p* = 0.702). These results indicate that the improvements seen in mood and physical activity were not significantly related to changes in serum BDNF levels. To determine if changes in mood (DASS-21 composite, GAD-7), sleep disturbance (GSAQ), and physical activity (IPAQ) predicted changes in serum BDNF across the intervention, an exploratory multiple linear regression analysis was performed. Changes in scores for each variable were entered as predictors, with changes in BDNF entered as the dependent variable. The overall model was statistically significant, *F*(4, 44) = 0.17, *p* = 0.954, and explained little variance in the changes in BDNF (*R*^2^ = 0.015; Adjusted *R*^2^ = −0.075). None of the individual predictors showed statistical significance (all *p* > 0.05). These results indicate that changes in psychological and behavioral measures were not associated with changes in serum BDNF during the intervention. Collinearity diagnostics showed acceptable variance inflation factors (VIF < 4), indicating no multicollinearity issues.

### 3.3. DASS-21 Depression Subscale Score

**Figure 3:**
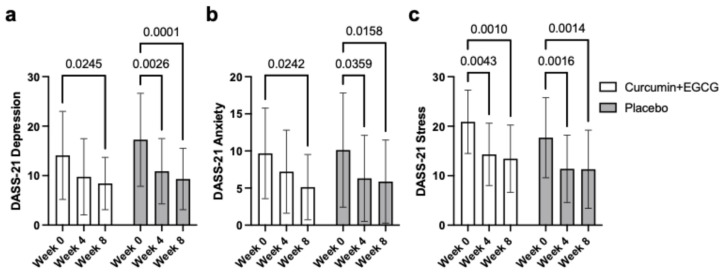
Mean outcomes for DASS-21 subscale and composite scores ((**a**) = Depression, (**b**) = Stress, and (**c**) = Anxiety) across time points (Weeks 0, 4, and 8) in both placebo and supplement groups. Values are presented as means ± standard deviation (SD). Illustrated are comparisons between the two groups across the four tests. DASS-21 = Depression, Anxiety, and Stress Scale—21 items.

The depression subscale scores showed a significant main effect of time across the three assessment points, *F*(2, 110) = 23.17, *p* < 0.001, partial h^2^ = 0.301, reflecting overall improvement in depressive symptoms (Figure 3a). With no significant group-by-time interaction, *F*(2, 110) = 0.72, *p* = 0.489, partial h^2^ = 0.013, it is evident that both the intervention and placebo groups had similar improvements. Post hoc comparisons revealed a similar trend found in the composite score, with significant reductions from Week 0 to Week 4 (MD = 5.35, *p* < 0.001) and from Week 0 to Week 8 (MD = 6.81, *p* < 0.001), but no significant difference from Week 4 to Week 8 (*p* = 0.241).

### 3.4. DASS-21 Anxiety Subscale Score

Similar to the depression subscale scores, subscale scores of anxiety also showed a significant main effect of time, *F*(2, 110) = 29.65, *p* < 0.001, partial h^2^ = 0.350, and no significant group by time interaction, *F*(2, 110) = 0.16, *p* = 0.857, partial h^2^ = 0.003, regardless of group assignment (Figure 3b). Following the same trend, Bonferroni-corrected pairwise comparisons found significant reductions from Week 0 to Week 4 (MD = 6.44, *p* < 0.001) as well as from Week 0 to Week 8 (MD = 6.91, *p* < 0.001) but no significant reductions between Week 4 and Week 8 (*p* = 1.000).

### 3.5. DASS-21 Stress Subscale Score

Stress subscale scores also demonstrated a significant main effect of time over the three time points, *F*(2, 110) = 21.36, *p* < 0.001, partial h^2^ = 0.280, with no significant group by time interaction observed, *F*(2, 110) = 0.79, *p* = 0.456, partial h^2^ = 0.014 (Figure 3c). The trend observed by Bonferroni-adjusted comparisons in depression and anxiety subscale scores was observed in stress as well, with significant decreases from Week 0 to Week 4 (MD = 3.15, *p* < 0.001) as well as Week 0 to Week 8 (MD = 4.41, *p* < 0.001), but no significant change between Week 4 and Week 8 (*p* = 0.073).

### 3.6. Generalized Anxiety Disorder Questionnaire (GAD-7)

**Figure 4:**
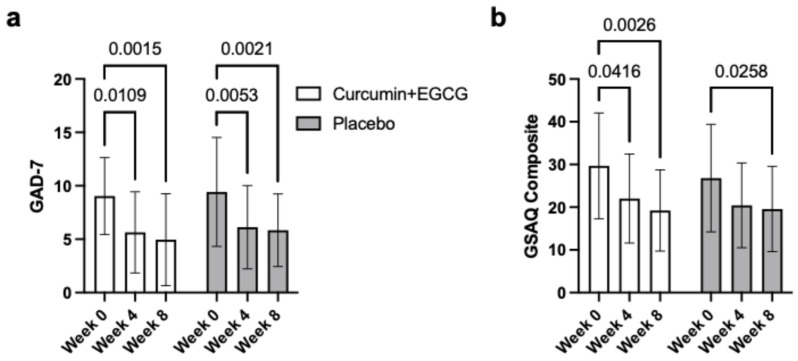
Mean GAD-7 and GSAQ scores across time points (Weeks 0, 4, and 8) by treatment group (Supplement vs. Placebo). Values are presented as means ± standard deviation (SD). (**a**): GAD-7 = Generalized Anxiety Disorder 7-item scale; (**b**): GSAQ = Global Sleep Assessment Questionnaire. The supplement group includes participants who received EGCG and curcumin; the placebo group received matched placebo capsules.

Anxiety (GAD-7) changes over the three time points were assessed using repeated measures ANOVA. There was a significant main effect of time, *F*(2, 108) = 26.18, *p* < 0.001, partial h^2^ = 0.327, indicating significant improvements in GAD-7 anxiety symptoms over the intervention duration (Figure 4a). Both the intervention and placebo groups followed a similar trend, revealing no significant interaction between group and time, *F*(2, 108) = 0.10, *p* = 0.902, as well as no significant between-group differences in overall anxiety scores, either *F*(1, 54) = 0.46, *p* = 0.503. Pairwise comparisons revealed similar trends to the DASS-21 subscale scores, with GAD-7 scores decreasing significantly from Week 0 to Week 4 (*p* < 0.001, MD = 3.35) and from Week 0 to Week 8 (*p* < 0.001, MD = 3.83) with no significant change from Week 4 to Week 8 (*p* = 0.741, MD = 0.49). This result is supported by a significant linear trend, *F*(1, 54) = 32.89, *p* < 0.001, partial h^2^ = 0.379, showcasing a steady decrease in anxiety over time and a significant quadratic trend, *F*(1, 54) = 12.48, *p* < 0.001, partial h^2^ = 0.188, suggesting improvements occurred during the earlier period of the intervention.

### 3.7. Global Sleep Assessment Questionnaire (GSAQ)

Changes in sleep disturbance (GSAQ) were examined using repeated measures ANOVA. A significant main effect of time was observed, *F*(2, 110) = 18.95, *p* < 0.001, partial h^2^ = 0.256, indicating a reduction in sleep disturbance across the intervention (Figure 4b). Pairwise comparisons showed significant decreases in aggregate GSAQ scores from Week 0 to Week 4 (*p* < 0.001, MD = 7.01) and from Week 0 to Week 8 (*p* < 0.001, MD = 8.83), with no significant difference between Week 4 and Week 8 (*p* = 0.516, MD = 1.83). Significant linear and quadratic trends were also revealed: *F*(1, 55) = 27.30, *p* < 0.001, partial h^2^ = 0.332; *F*(1, 55) = 5.17, *p* = 0.027, partial h^2^ = 0.086, respectively. However, no significant group-by-time interaction, *F*(2, 110) = 0.56, *p* = 0.570, or overall group difference, *F*(1, 55) = 0.35, *p* = 0.558, was observed, indicating comparable improvements in sleep quality across both groups.

### 3.8. International Physical Activity Questionnaire (IPAQ)

**Figure 5:**
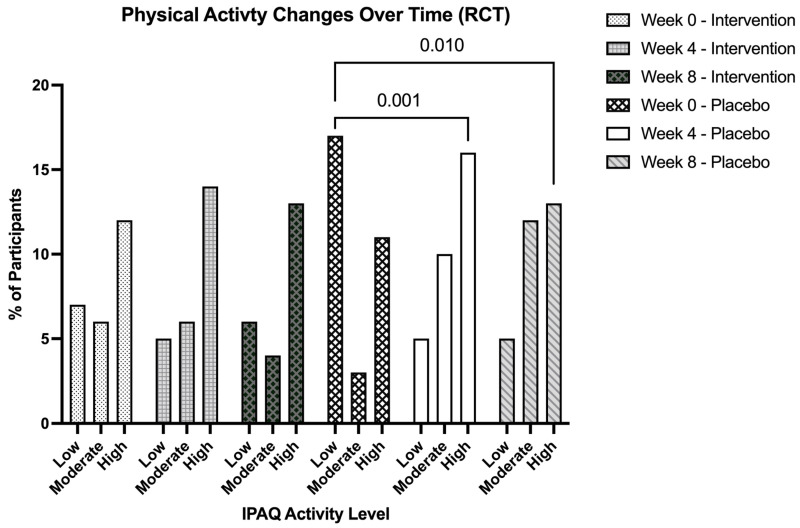
Percentage distribution of International Physical Activity Questionnaire (IPAQ) activity levels (low, moderate, high) across time points (Weeks 0, 4, and 8) by treatment group. Activity level categories were derived from MET-minutes per week according to IPAQ scoring protocols. Values represent the percentage of participants in each category at each time point.

To assess changes in physical activity levels between the two groups over the 8-week intervention, a Friedman test was conducted using the IPAQ standardized levels of low, moderate, and high. The placebo group demonstrated a significant overall change in physical activity levels over time, c^2^ (2) = 11.31, *p* = 0.003. Using post hoc Wilcoxon signed-ranked tests with Bonferroni correction (adjusted α = 0.017), significant increases in activity were observed between Week 0 and Week 4 (Z = −3.22, *p* = 0.001) and also between Week 0 and Week 8 (Z = −2.58, *p* = 0.010), but no significant change between Week 4 and Week 8 (Z = −0.44, *p* = 0.660) of the placebo group. This finding points to increases in physical activity in the initial four weeks of the 8-week intervention (Figure 5). Interestingly, the intervention group displayed more stable physical activity levels, exhibiting no significant changes over time c^2^ (2) = 1.09, *p* = 0.581, with all Wilcoxon comparisons remaining non-significant (all *p* > 0.35). To explore the direction of these categorical shifts, cross-tabulation analyses were employed.

The first half of the intervention, from Week 0 to Week 4, saw 42.9% of intervention participants initially classified as “low” transitioning to “high”, with 50% of placebo participants in the “low” category transitioning to “moderate” or “high” activity. Pearson chi-square tests support the finding that both groups showed significant distribution changes over this time, c^2^ (4) = 10.99, *p* = 0.027 (intervention) and c^2^ (4) = 13.12, *p* = 0.011 (placebo). When examining the duration in full, Week 0 to Week 8 revealed 50% of those initially in the “low” category of the intervention group transitioned to higher activity levels, while 76.5% of “low” participants of the placebo group transitioned to “moderate” or “high.” Neither of these changes, however, showed significance for either group (intervention: c^2^ (4) = 7.69, *p* = 0.104; placebo: c^2^ (4) = 6.63, *p* = 0.157). Examining the latter half of the intervention (Week 4 to Week 8), we saw 60% of participants in the intervention group classified as the “moderate” category transition to “high.” Overall chi-square tests were not significant, yet it should be noted that both groups demonstrated significant linear-by-linear associations (intervention: c^2^ (1) = 6.33, *p* = 0.012; placebo: c^2^ (1) = 4.96, *p* = 0.026), suggesting meaningful upward trends.

### 3.9. Dietary Intake

Repeated measures ANOVA indicated no significant within-group changes over time and no significant group-by-time interactions for total energy, fat, carbohydrate, protein, fiber, or sugar intake between the intervention and placebo groups (all *p* > 0.05). There was, however, a significant main effect of group observed for sugar intake (*p =* 0.030). Specifically, total energy intake remained stable throughout the study, with no significant effects of group (*p* = 0.623), time (*p* = 0.244), and group by time interaction (*p* = 0.644), indicating no meaningful change in caloric intake over the intervention period (Table 2). Similar non-significant patterns were identified for fat (group *p* = 0.929; time *p* = 0.419; group × time *p* = 0.985), carbohydrate (group *p* = 0.708; time *p* = 0.241; group × time *p* = 0.801), and fiber (group *p* = 0.530; time *p* = 0.500; group × time *p* = 0.708). Protein showed a modest increase in the placebo group at Week 8, but no significant main effects or interactions were observed (group *p* = 0.288; time *p* = 0.533; group × time *p* = 0.556). In contrast, sugar intake was consistently higher in the intervention group at both assessment points with a significant group effect (*p* = 0.030) and no significant time (*p* = 0.775) or group × time interaction (*p* = 0.911), suggesting stable intake across the trial.

**Figure 6:**
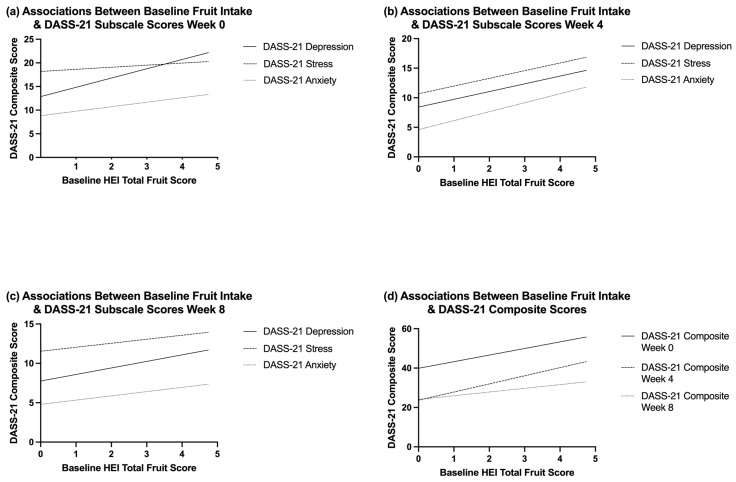
Linear trend lines showing the associations between baseline Healthy Eating Index (HEI) total fruit scores and DASS-21 subscale scores at Week 0 (**a**), Week 4 (**b**), Week 8 (**c**), and composite scores across time (**d**). Lines illustrate the direction of association and do not imply causality.

Accounting for background dietary polyphenol consumption, baseline Healthy Eating Index (HEI) scores for total fruit and total vegetable intake were included as covariates in repeated-measures ANCOVA models examining DASS-21 subscale and composite outcomes. Baseline fruit intake was significantly associated with depression scores across time *F*(1, 53) = 6.12, *p* = 0.017) (Figure 6). Fruit intake was also significantly associated with stress (*b* = 1.572, *p* = 0.019) and anxiety (*b* = 1.437, *p* = 0.015) at Week 4 (Figure 6b). Significant associations were also observed between fruit intake and DASS-21 composite scores at both Week 0 (*b* = 4.669, *p* = 0.033) and Week 4 (*b* = 4.324, *p* = 0.010) (Figure 6d). Higher vegetable intake was associated with lower stress (*b* = 1.486, *p* = 0.021) and composite DASS-21 scores (*b* = 2.764, *p* = 0.024) at Week 8 only. Change scores for fruit and vegetable intake were additionally examined to assess whether dietary improvements influenced mood outcomes. No significant interactions were observed between time and changes in fruit and vegetable intake, nor were there significant between-subjects effects for either (all *p* > 0.05). These sensitivity analyses indicate that short-term increases in fruit or vegetable consumption were not significantly associated with changes in overall mood disorder symptomology over the 8-week trial. This suggests that habitual dietary intake may have had more of an impact than short-term dietary modification.

### 3.10. Sugar and Mental Health

**Figure 7:**
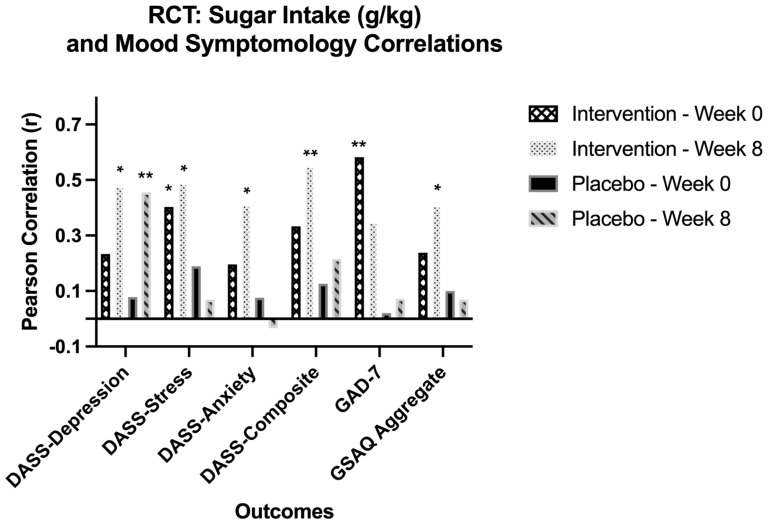
A Pearson correlation showing associations between dietary sugar intake (g/kg body weight) and mood disturbance outcomes across time points (Weeks 0 and 8) in the randomized controlled trial (RCT). Significance (* *p* < 0.05, ** *p* < 0.01).

Pearson correlation analyses were performed to assess relationships between sugar intake normalized to body weight (g/kg) and mental health outcomes (Figure 7). At baseline, significant positive associations were observed in the intervention group between sugar intake and DASS-21 stress (r = 0.403, *p* = 0.034) and GAD-7 (r = 0.582, *p* = 0.001), whereas no associations were detected for subscale depression, anxiety, sleep disturbance, or DASS-21 composite scores (all *p* > 0.05). No significant correlations were identified in the placebo group at baseline (all *p* > 0.05). By Week 8, associations were detected in the intervention group, with higher sugar intake significantly correlated with DASS-21 depression (r = 0.474, *p* = 0.017), stress (r = 0.487, *p* = 0.014), anxiety (r = 0.408, *p* = 0.043), GSAQ sleep disturbance (r = 0.405, *p* = 0.044), and DASS-21 composite scores (r = 0.547, *p* = 0.005). GAD-7 scores showed a positive but non-significant trend. (r = 0.345, *p* = 0.091). In contrast, the placebo group showed sugar intake to be significantly associated only with DASS-21 depressive symptoms (r = 0.455, *p* = 0.009), with no other mental health outcome showing significant relationships (all *p* > 0.05). Based on these findings, sugar intake became more strongly associated with adverse mood and sleep outcomes over time in the intervention group, whereas baseline associations were limited.

### 3.11. Comprehensive Metabolic Panel

Repeated measures ANOVA was used to explore changes in comprehensive metabolic panel markers (CMP) across the 8-week intervention and between groups (intervention vs. placebo). A significant main effect of time was revealed for glucose, sodium, potassium, and chloride (all *p* < 0.05), indicating these markers decreased over time regardless of group assignment (Table 3). Stable liver enzyme concentrations resulted in no significant main or interaction effects for ALT or AST (all *p* > 0.05). At the levels observed over the eight weeks, both remain in the normal reference range of ALT (7–56 U/L) and AST (0–35 U/L) [57]. No significant group or group-by-time effects were observed for any of the tested markers (all *p* > 0.05). The indicated results for CMP markers showed some improvement over time, but the intervention did not result in significantly different effects compared to placebo.

### 3.12. Adherence

Adherence to the supplement protocol was calculated based on the percentage of days each participant reported consuming their assigned pills over the 8-week trial (56 days). The mean adherence rate was high, 94.39% (SD = 6.99), with individual adherence ranging from 69.64% to 100%. Independent samples t-tests revealed no significant differences in adherence between the intervention (M = 94.14%, SD = 7.19) and placebo groups (M = 94.59%, SD = 6.95), *t*(55) = −0.236, *p* = 0.814. To ensure that the percentages of adherence did not affect the primary outcomes, Pearson correlations were conducted between adherence percentage and Week 8 scores on DASS-21, GAD-7, GSAQ, and serum BDNF concentrations. These tests revealed no significant correlations between adherence and depressive symptoms (*r* = 0.078, *p* = 0.563), anxiety (*r* = −0.169, *p* = 0.210), stress (*r* = −0.005, *p* = 0.971), GAD-7 (*r* = −0.079, *p* = 0.557), GSAQ (*r* = −0.024, *p* = 0.862), or total DASS-21 composite scores (*r* = −0.031, *p* = 0.820). Changes in serum BDNF concentrations were also found to be unrelated to adherence at Week 8 (*r* = 0.189, *p* = 0.172). Overall, the results indicate that adherence was uniformly high across both groups, showing that differences in adherence did not meaningfully impact primary outcomes during the study period.

## 4. Discussion

This randomized, double-blind, placebo-controlled trial looked at the impact of supplementation with both EGCG and curcumin on mood disorder symptomology and serum BDNF concentrations in a moderate to severely depressed adult population. Significant improvements were observed in mood-related outcomes, including the DASS-21 composite and subscale scores (depression, anxiety, and stress), as well as GAD-7 scores (all *p* < 0.001), alongside improvements in sleep (*p* < 0.001) and physical activity (*p* < 0.01) across all participants. These improvements occurred independent of supplementation, as no significant differences were observed between the intervention and placebo groups. Correlation analyses revealed significant positive associations between dietary sugar intake (g/kg body weight) and mood symptoms at Week 8 within the intervention group, underscoring the potential influence of dietary factors on mental health outcomes. To account for dietary polyphenol intake, the Healthy Eating Index (HEI) fruit and vegetable scores were included as covariates. Baseline fruit intake was positively associated with depression scores across time, as well as stress, anxiety, and DASS-21 Composite Scores at select time points. Given this, however, changes in fruit and vegetable intake during the intervention were not significantly related to changes in mood symptoms, suggesting that observed improvements were not attributable to concurrent dietary modifications. The following paragraphs will discuss these findings in greater detail.

The most notable finding herein was significant improvements in depression, anxiety, and stress (DASS-21, GAD-7) across time, which is consistent with previous literature [25,30]. Since there was no significant group-by-time interaction regardless of the mood domain, this could suggest the potential placebo effect or both groups benefiting from monitoring and structured participation, supported by the Hawthorne Effect [58]. Engagement in scheduled assessments, repeated self-reporting of mood, and regular contact with the research staff can enhance self-awareness and promote behavioral adjustments regardless of active treatment.

Interestingly, post hoc Bonferroni-adjusted pairwise comparisons revealed a consistent pattern across DASS-21 and GAD-7 outcomes, showing significant symptom improvements during the first half of the intervention (Week 0 to Week 4), but no significant changes from Week 4 to Week 8. Following this baseline to Week 4, early intervention trend of significance was improvements in sleep disturbance. There was a significant decrease in both groups over time, but no differences between the intervention and placebo groups. Despite prior evidence linking green tea bioactives to improved sleep [59], the observed findings suggest the improvements were likely related to study participation rather than supplement-specific effects. A similar pattern was observed for physical activity, as IPAQ categories showed significant increases in the placebo group from Week 0 to Weeks 4 and 8, while no significant changes appeared in the intervention group. The early pattern of symptom improvement across both groups, coupled with improvements in sleep and physical activity, supports the possibility of behavioral activation during the first half of the intervention. Behavioral activation centers around reinforcement models and scheduled rewards, which increase adaptive behaviors with positive reinforcement [60]. The use of daily adherence messages and scheduled assessments could have acted as these scheduled rewards. The observed phenomenon also aligns with behavior change models like the Health Action Process Approach (HAPA) [61], which helps to bridge the understanding between how determinant factors like expectations of desired outcomes and self-efficacy can lead to behavior change.

Future studies could incorporate methodological approaches that more effectively distinguish intervention-specific effects from participation-related influences. This could include evaluating expectancy beliefs, implementing a run-in phase prior to randomization, using objective behavioral measures, or alternative control conditions like active or delayed-intervention comparators.

When exploring dietary impacts on mood outcomes, baseline sugar intake was positively correlated with stress and anxiety in the intervention group only. By Week 8, higher sugar intake in this group was strongly associated with worse depression, stress, anxiety, overall mood, and sleep disturbance, with a similar but weaker pattern observed for anxiety. In the placebo group, sugar intake was associated only with depressive symptoms at Week 8. The mechanisms by which sugar intake can impact mood are varied. Associations between carbohydrate consumption and increased circulation of inflammatory markers have been observed, as well as hypoglycemia from an exaggerated insulin response. All of which, through hormonal influences, could potentially alter mood [62]. There also seems to be strong evidence for the influence of sugar in reducing the activity of the hypothalamic–pituitary–adrenal axis, which regulates the body’s response to stress and controls mood [63]. Other studies have noted potential links between sugar and its metabolism and axonal degeneration, neuronal activity, neurotransmitter imbalance, impaired neurogenesis, and altered brain plasticity [64].

These analyses were exploratory in nature and involved multiple comparisons. Therefore, findings should be interpreted cautiously. While prior literature has linked sugar intake with adverse mental health outcomes [65,66], the present study was not powered to evaluate dietary predictors of mood disturbance. These associations should be considered hypothesis-generating and warrant confirmation in larger trials designed specifically to examine dietary impact on mental health. Total energy and macronutrient intake showed no significant intervention effects, with higher sugar intake in the intervention group showing moderate effect size trends and a positive association with depression and overall mood disturbance at Week 8. Future research specifically designed to evaluate dietary patterns under controlled conditions may help clarify the directionality and strength of these relationships. Randomized controlled trials examining sugar reductions or broader dietary pattern modification, paired with metabolic and inflammatory biomarkers, could provide stronger evidence regarding the role of dietary intake in mood regulation.

Baseline fruit and vegetable intake was examined to account for background polyphenol exposure. Higher fruit intake was positively associated with mood disturbance at earlier time points, while vegetable intake was significantly associated with stress and overall mood at Week 8. Although prior studies often report inverse associations between fruit intake and mood disturbance, they look at risk rather than current depressive symptomology [67]. Importantly, this finding should not be interpreted as evidence that fruit intake exacerbates mood disturbance. Rather, the observed association may reflect health-seeking or compensatory behaviors in those experiencing greater mood disturbance, having already adopted healthier eating patterns at baseline. These associations were independent of group, and changes in fruit and vegetable intake over the intervention were not related to mood improvements, which suggests habitual intake played a larger role than short-term dietary changes. Collectively, these findings suggest that short-term improvements in mood and sleep were driven primarily by early study engagement and monitoring effects. Habitual dietary patterns, specifically higher sugar intake and baseline fruit and vegetable consumption, appear to be more impactful in modulating mood and sleep compared to the EGCG and curcumin supplementation alone.

We also discovered that serum BDNF concentrations did increase over time in both groups, but not to a statistically significant amount, appearing not to be driven by supplementation. Serum BDNF is known to exhibit considerable biological variability and may be influenced by behavioral and environmental factors such as sleep, physical activity, and stress [68]. Exploratory analyses revealed that changes in mood (DASS-21, GAD-7), sleep (GSAQ), and physical activity (IPAQ) were not significant predictors of changes in serum BDNF. These findings indicate that, given the intervention duration, dosage, and characteristics of the study population, measurable alterations in serum BDNF may have been limited.

### Limitations

There are limitations with the current study that should be noted. First, there would be the short intervention duration. Longitudinal effects might have been observed with an intervention lasting longer than 8 weeks and might have allowed for significant between-group differences. There was also the absence of long-term follow-up beyond the 8-week supplementation period; therefore, the persistence of observed effects after discontinuation of the supplements could not be evaluated. Future studies incorporating post-intervention follow-up assessments (e.g., 12–16 weeks or longer) would help determine whether mood-related effects are sustained over time.

Serum BDNF was assessed only at baseline and Week 8, which may have limited the ability to capture dynamic neurobiological changes during the intervention period. Given the biological variability of peripheral BDNF and its susceptibility to behavioral and environmental influences, more frequent biomarker assessments may provide greater sensitivity in future trials.

The sample size was modest, and attrition may have reduced statistical power. Outcomes like mood, physical activity, and dietary intake were self-reported, introducing potential bias due to underreporting, social desirability, and recall error. As described in the Methods, participants were classified based on DASS-21 symptom severity rather than formal psychiatric diagnoses, and information regarding duration of symptoms or prior treatment history was not assessed. Therefore, findings may not generalize to individuals with clinically diagnosed mood disorders. Additionally, the predominance of female participants may restrict applicability to more gender-balanced or clinically diverse populations.

## 5. Conclusions

In conclusion, findings from this trial indicate that EGCG and curcumin supplementation did not produce significant improvements in mood disturbance or serum BDNF beyond those observed with placebo. Although reductions in mood symptoms and improvements in subjective sleep were observed over the 8-week period, these changes were not specific to the intervention group. Furthermore, the observed associations between dietary sugar intake and depressive symptoms and total mood disturbance at Week 8 emphasize the clinical importance of dietary influences on mental health outcomes. These findings should be interpreted cautiously and warrant further investigation in controlled trials.

## Figures and Tables

**Table 1:** Baseline characteristics of RCT study participants (*n* = 60).

Characteristic		Total	Intervention *n* = 28	Placebo *n* = 32	*p*
Gender	Female	38 (63.3)	18 (64.3)	20 (62.5)	0.886
	Male	22 (36.7)	10 (35.7)	12 (37.5)	
Ethnicity	White	35 (58.3)	17 (60.7)	18 (56.3)	0.726
	Other	25 (41.7)	11 (39.3)	14 (43.8)	
Age	mean (s.d.)	27.4 (5.2)			0.235
	18–24	17 (28.3)	10 (35.7)	7 (21.9)	
	25+	43 (71.7)	18 (64.3)	25 (78.1)	
BMI	mean (s.d.)	26.6 (5.5)			0.713
	18.5–24.9	41 (41.8)	27 (45)	14 (36.8)	
	25–29.9	35 (35.7)	20 (33.3)	15 (39.5)	
	>30	22 (22.4)	13 (21.7)	9 (23.7)	
DASS-21 Composite (Baseline-Adjusted to DASS-42 Scale)	Mean (s.d.)	44.7 (20.3)	44.36 (17.9)	45.06 (22.5)	0.893

**Table 2:** Means and between-group comparisons for dietary intake variables in the RCT (*n* = 57). Significance (* *p* < 0.05).

	Intervention	Placebo	Intervention	Placebo			
Variable	Week 0	Week 0	Week 8	Week 8	Group	Time	Group × Time
	Mean (SD)	Mean (SD)	Mean (SD)	Mean (SD)	*p*	*p*	*p*
Energy (kcal)	2012.8 (522.3)	1904.9 (728.6)	1870.9 (517.8)	1844.7 (593.1)	0.623	0.244	0.644
Fat (g)	80.4 (30.1)	80.9 (41.1)	75.8 (22.7)	76.6 (29.1)	0.929	0.419	0.985
Protein (g)	93.4 (39.3)	80.9 (46.5)	89.3 (36.1)	82.5 (28.6)	0.288	0.533	0.556
Carbohydrate (g)	225.0 (63.0)	216.1 (81.3)	208.4 (69.3)	204.6 (75.7)	0.708	0.241	0.801
Dietary Fiber (g)	19.1 (6.4)	18.4 (8.9)	18.07 (6.2)	16.5 (9.1)	0.530	0.500	0.708
Sugars (g)	74.5 (27.5)	59.1 (30.1)	76.1 (33.8)	61.9 (32.9)	* 0.030	0.775	0.911

**Table 3:** Estimated marginal means of key metabolic biomarkers by group across RCT study visits (*n* = 59). Significance (* *p* < 0.05, *** *p* < 0.001).

	Intervention		Placebo				
Variable	Week 0	Week 8	Week 0	Week 8	Group	Time	Group × Time
	Mean (SD)	Mean (SD)	Mean (SD)	Mean (SD)	*p*	*p*	*p*
Glucose	93.4 (14.8)	86.7 (7.8)	94.2 (11.6)	88.3 (7.3)	0.522	<0.001 ***	0.940
Sodium	147.3 (13.1)	139.5 (1.9)	147.1 (11.8)	138.8 (1.5)	0.835	<0.001 ***	0.864
Potassium	4.6 (0.53)	4.4 (0.29)	4.6 (0.88)	4.3 (0.3)	0.623	0.018 *	0.486
Chloride	110.7 (9.8)	105.1 (1.6)	110.7 (8.2)	104.6 (2.4)	0.868	>0.001 ***	0.784
ALT	15.8 (7.8)	15.9 (11.3)	16.1 (9.1)	15.4 (9.5)	0.937	0.853	0.803
AST	19.8 (8.3)	17.2 (6)	18.5 (3.8)	18.9 (13.7)	0.9	0.445	0.349

## Data Availability

The original contributions presented in this study are included in the article. Further inquiries can be directed to the corresponding author.

## Abbreviations

The following abbreviations are used in this manuscript:

BDNF	Brain-Derived Neurotrophic Factor
EGCG	Epigallocatechin-3-gallate
DASS-21	Depression, Anxiety, Stress Scale-21
GAD-7	Generalized Anxiety Disorder-7
GSAQ	Global Sleep Assessment Questionnaire
IPAQ	International Physical Activity Questionnaire
CAM	Complementary and Alternative Medicine
MDD	Major Depressive Disorder
ALT	Alanine Transaminase
AST	Aspartate Aminotransferase
ANOVA	Analysis of Variance
MANOVA	Multivariate Analysis of Variance
WHO	World Health Organization
HEI	Healthy Eating Index
